# How much time do health services spend on antenatal care? Implications for the introduction of the focused antenatal care model in Tanzania

**DOI:** 10.1186/1471-2393-6-22

**Published:** 2006-06-23

**Authors:** Claudia von Both, Steffen Fleβa, Ahmad Makuwani, Rose Mpembeni, Albrecht Jahn

**Affiliations:** 1 Department of Tropical Hygiene and Public Health, Ruprecht-Karls-University, Im Neuenheimer Feld 324, 69120 Heidelberg, Germany; 2 Department of Health Care Management, Faculty of Law and Economics, University of Greifswald, Friedrich-Loeffler-Str. 70, D-17487 Greifswald, Germany; 3 School of Public Health & Social Sciences, Muhimbili University College of Health Sciences, Daressalaam, Tanzania

## Abstract

**Background:**

Antenatal care (ANC) is a widely used strategy to improve the health of pregnant women and to encourage skilled care during childbirth. In 2002, the Ministry of Health of the United Republic of Tanzania developed a national adaptation plan based on the new model of the World Health Organisation (WHO). In this study we assess the time health workers currently spent on providing ANC services and compare it to the requirements anticipated for the new ANC model in order to identify the implications of Focused ANC on health care providers' workload.

**Methods:**

Health workers in four dispensaries in Mtwara Urban District, Southern Tanzania, were observed while providing routine ANC. The time used for the overall activity as well as for the different, specific components of 71 ANC service provisions was measured in detail; 28 of these were first visits and 43 revisits. Standard time requirements for the provision of focused ANC were assessed through simulated consultations based on the new guidelines.

**Results:**

The average time health workers currently spend for providing ANC service to a first visit client was found to be 15 minutes; the provision of ANC according to the focused ANC model was assessed to be 46 minutes. For a revisiting client the difference between current practise and the anticipated standard of the new model was 27 minutes (9 vs. 36 min.). The major discrepancy between the two procedures was related to counselling. On average a first visit client was counselled for 1:30 minutes, while counselling in revisiting clients did hardly take place at all. The simulation of focused ANC revealed that proper counselling would take about 15 minutes per visit.

**Conclusion:**

While the introduction of focused ANC has the potential to improve the health of pregnant women and to raise the number of births attended by skilled staff in Tanzania, it may need additional investment in human resources. The generally anticipated saving effect of the new model through the reduction of routine consultations may not materialise because the number of consultations is already low in Tanzania with a median of only 4 visits per pregnancy. Special attention needs to be given to counselling attitudes and skills during the training for Focused ANC as this component is identified as the major difference between old practise and the new model. Our estimated requirement of 46 minutes per first visit consultation matches well with the WHO estimate of 40 minutes.

## Background

The World Health Report 2005 calls for "Realizing the Potential of Antenatal Care" [1]. While antenatal care interventions, in and of themselves, cannot be expected to have a major impact on maternal mortality, their purpose is to improve maternal and perinatal health, 'this being an end in itself and necessary for improving the health and survival of infants' [2]. In addition, the potential of ANC as a line of action to increase the rate of births attended by skilled health staff, and its value as entry point for other health programmes such as malaria, TB, nutrition and HIV/AIDS is now better understood and applied [1-4].

The concept of the new ANC model, as promoted by WHO, reflects this new understanding of the role of ANC [5]. The number of visits for those, identified not to be at high risk, is reduced to four. Each of these visits consists of a well defined set of activities related to three equally important general areas, namely (1) 'screening for conditions likely to increase adverse outcomes', (2) 'providing therapeutic interventions known to be beneficial', and (3) 'educating pregnant women about planning for a safe birth, emergencies during pregnancy and how to deal with these [5]. It has been highlighted that since the number of visits is reduced to four in the basic component, 'sufficient time must be made during each visit for discussion of the pregnancy and related issues with the patient' [5]. The emphasis on communication in the new antenatal care model was appreciated by women and health care providers alike [6].

It is estimated that 21,000 mothers died in Tanzania due to complications in pregnancy and delivery in the year 2000 [7] despite a high coverage of ANC of 98% [8]. The median number of antenatal consultations is 3.9 [9]. ANC is provided by health workers with 2 to 4 years of professional training. Quality assessments showed that less than half of all women get information about pregnancy complications, receive iron tablets, and have access to anti-malarial medicine. About 44 % of all women in Tanzania deliver at a health facility [8]. These data suggests that the potential benefits of ANC are not yet exploited.

Since 2002, the Ministry of Health of the United Republic of Tanzania promoted a new model of ANC, called 'Focused ANC', a national adaptation of the new WHO concept. The elements of the new model comprise the early detection and management of disease/abnormality, counselling on health promotion, counselling on birth preparedness, complication readiness and the development of an individual birth plan [10]. Counselling and health education have therefore become a major strategy to improve maternal health and, in particular, to increase the proportion of skilled delivery. The new concept is in the process of implementation. Table 1 shows the proposed content of each of the routine ANC visits.

**Table 1:** Focused antenatal care model Tanzania (checklist)

**Focused antenatal care **				
**Checklist**				
**Parameter**	**First visit **(< 16 weeks)	**Second visit **20–24 weeks	**Third visit **28–32 weeks	**Fourth visit **(36 weeks)
**1. Registration**				

**2. History taking**				
Personal history				
Family history				
Social history				
Past medical/surgical history				
History of complaints in current pregnancy				

**3. Examination**				
Head to toe (whole body)				
Pallor				
Oedema (other than ankle-specify)				
Breast				
Lungs and heart				

**4. Observation and clinical investigation**				
Temperature				
Pulse				
Blood pressure				
Weight				

**5. Obstetric complications**				
Fundal height				
Foetal presentation and engagement				
Foetal heart sound				

**6. Pelvic (vaginal) examination**				
Soft tissue assessment				
Bony pelvic assessment				

**7. Laboratory investigations**				
**Blood: **				
Haemoglobin				
Grouping and rhesus factor				
RPR				
HIV testing				
**Urine:**				
Protein, sugar, acetone				

**8. Drug administration and immunization**				
Iron				
Folic acid				
Antimalarials (Fansidar 3 tablets)				
Tetanus toxoid				

**9. Client Education and counselling (for the couple)**				
Process of pregnancy and its complications				
Diet and nutrition				
Rest and exercise in pregnancy				
Personal hygiene				
Danger signs in pregnancy				
Use of drugs in pregnancy				
Effects of STI/HIV/AIDS				
Voluntary counselling and testing for HIV				
Care of breasts and breast feeding				
Symptoms/signs of labour				
Plans of delivery (emergency preparedness, place of delivery, transportation, financial arrangements)				
Plans for postpartum care				
Family planning				
Harmful habits (e.g. smoking, drug abuse, alcoholism)				
Schedule of return visit				

This paper describes the current performance of ANC in dispensaries in South Tanzania with special emphasis on an assessment of the time health care providers spend on the different activities of ANC. The current practice is compared with standard times of the new focused ANC model.

## Methods

### Study area

The study has been carried out in the south-eastern part of Tanzania in Mtwara Urban District; one of the five districts of Mtwara Region. This region belongs to one of the less developed parts of Tanzania. About 90 % of its population live in rural areas on subsistence farming. Mtwara Urban District consists of Mtwara town and some adjacent villages. The district comprises a population of 92,602, of which 45,122 are male and 47,480 are female. About 60 % of the population live in urban and 40 % in rural areas [11]. The crude birth rate per 1,000 of Mtwara Region is 41 [8].

In Mtwara Urban District, eight governmental dispensaries and one hospital offer antenatal care services. The hospital (Ligula) is the regional hospital and also serves as district hospital for Mtwara Urban and Mtwara Rural District.

### Study design

The study was descriptive and used primarily quantitative methods of data collection. The data collection was carried out during a period of four weeks from April to May 2004. Four of the eight governmental dispensaries in Mtwara Urban District were selected in consultation with the Health Department of the regional and local authorities: the Mother and Child Health (MCH) clinic attached to the Ligula Regional Hospital in Mtwara town, the two biggest dispensaries in the Mtwara Urban District, both are currently in the process of being upgraded to a Health Centre and one dispensary representative of a more rural area. The dispensaries were visited at randomly chosen days and times. Twenty-five visits were made to the dispensaries. Thirteen different formally trained health workers (nurses, midwives and mother-and-child health aids) were observed as they provided ANC services.

The staff of the dispensaries had, at the time of the study, already received basic training in the Focused Antenatal Care model. They knew that in the basic component the number of visits is reduced to four. They were aware of the timing of the visits, of the drugs and immunizations to be provided and the subjects of counselling. Further in depth training was in the planning stage [12].

The leading researcher and one assistant, familiar with the Swahili language, observed the process of ANC service provision and clocked the time used with a stopwatch. A total of 71 visits were observed, comprising all antenatal consultations encountered during the visits. The age range was 16 to 44 years with a majority of multiparous mothers. Data was separately collected for first visit (28) and revisit clients (43). A revisit was defined as any other visit than the first.

The different steps of an ANC visit as shown in table 1 were defined as (1) registration, (2) history taking, (3) examination (including steps 3–6 of the checklist), (4) drug administration and (5) immunization, and (6) health education and counselling. Other activities like the (7) documentation of findings and (8) the time of welcoming the client have in some cases also been assessed. Time was only registered as counselling when the service provider communicated with the client and was not at the same time primarily involved with other activities. Therefore, information regarding the status of the women and her current pregnancy given during examination was not recorded as counselling. As the respective time of each step of service provision was separately measured with the stopwatch, the total time of the visit was calculated as the sum of the different steps observed. While doing this time study the general structure of service provision was observed.

The standard times of the new focused ANC model were defined by a series of 10 simulations of ANC service delivery based on the guidelines of the new model. The simulations were done during a focused ANC training under the supervision of one of the authors (AM). In addition, a more detailed analysis of the time required for client education and counselling and counselling on drugs was undertaken. For this purpose one role-play was performed and the time used was clocked with a stopwatch. The provider side was simulated by one of the authors (AM). For the role of the client, a pregnant, semi-urban woman with little education was chosen. The simulated counselling processes followed exactly the guidelines of the new focused ANC model, as listed in table 1 'Client Education and Counselling' [10]. This study focuses on the time providers spend currently on ANC or would need to spend for fully implementing the focused ANC concept.

### Data analysis

The collected data on the current performance of ANC was entered in a Microsoft EXCEL 2000 roster assessing the arithmetic mean, the minimum and maximum, and the range of the total time of antenatal first and revisit as well as of each previously defined step of the service provision process. These results were compared with standard times.

## Results

### Structure of ANC service provision

Services start generally with a general health education presentation about subjects such as breast-feeding or HIV, addressing all gathered clients and patients. Antenatal clients, family planning clients and other patients are encouraged to come to the dispensary early in the morning in order to attend these sessions. However, there may be a considerable time lag until actual antenatal consultations start. The official working hours are from 8 am till 3.30 pm. The hospital-based MCH clinic in Mtwara and one of the bigger dispensaries offer ANC services all day, the others offer them for 2 to 3 hours per working day. In half of the observed antenatal clinics, all clients and patients were attended prior to 12 am. The others finished in the early afternoon, while in 3 of the 25 observed clinics, antenatal consultations continued till 3 pm.

The organisation of ANC service varied among the dispensaries. In most dispensaries, one service provider was in charge of ANC and performed all the steps, often including immunization. In one dispensary a production line was used and each step was performed by another service provider.

### Time assessment of current ANC service performance

#### ANC first visit

A total of 28 antenatal first visits were observed. Basic activities such as registration, history taking, examination and drug administration were always performed. Immunization was documented 18 times. Specific individual counselling was performed only in 8 (30%) out of 28 consultations. The average time used for a first ANC visit was 12:20 minutes, with a minimum of 8:00 minutes and a maximum of 20:40 minutes.

Table 2 presents the time used for each step of ANC. Activities are listed according to the flow of service provision. History taking was the longest step followed by examination, registration, immunization, counselling, and drug administration. Counselling took on average 1:30 minutes. Key topics covered were the danger and prevention of anaemia and malaria as well as issues of hygiene and breastfeeding.

**Table 2:** Time (in min) for activities in ANC 1^st ^visit; current practice

**ANC FIRST VISIT**	**no. of observations**	**mean**	**minimum**	**maximum**
Registration	26	02:10	01:00	03:30
History taking	28	04:20	02:30	08:20
Examination	28	03:40	02:00	07:20
Drug administration	28	01:00	00:30	03:00
Health education and counselling	8	01:30	00:20	02:10
Immunization	18	01:40	00:20	03:30
				
Total ANC contact time *	28	12:20	08:00	20:40

* The total contact time is not the sum of the average times of each step, but calculated as the mean of the total time of the observed visits. It differs from the sum of the average times because the number of observations per service step varies.

The overall contact time for a first ANC visit was 15:20 minutes, including activities such as the documentation of findings (mean of 2:00 minutes), and the time spent for welcoming the client (mean of 1:00 minute).

#### Antenatal revisit

In total 43 antenatal revisits were observed, but the number of observations for each step in the ANC provision varied widely as shown in table 3. Specific counselling and preparation of individual births plans did not take place at all during our observations. As mentioned in *the methodology section*, counselling was only documented as such when providing health education or developing a birth plan was the primary activity of the provider. The average time of an ANC revisit was 6:30 minutes. The minimum was 3:20 minutes; the maximum recorded was 13:10 minutes. Table 3 presents the time used for the specific ANC activities. Here the overall contact time was only 9:00 minutes.

**Table 3:** Time (in min) used for ANC activities during re-visits

**ANC REVISIT**	**no. of observations**	**mean**	**minimum**	**maximum**
Registration	40	01:30	00:20	03:20
History taking	18	01:20	00:30	04:20
Examination	43	03:00	01:30	06:00
Drug administration	39	01:40	00:20	04:00
Health education and counselling	0	00:00	00:00	00:00
Immunization	6	01:00	00:20	02:50
				
Total ANC contact time *	43	06:30	03:20	13:10

* The total contact time is not the sum of the average times of each step, but calculated as the mean of the total time of the observed visits. It differs from the sum of the average times because the number of observations per service step varies.

#### Time assessment of the new focused ANC

According to our simulations of ANC consultations following the new focused ANC model the following standard times were observed: Registration for a first antenatal visit 5 minutes, history taking 10 minutes, a thorough examination 8 minutes and the drug administration 3 minutes, counselling 15 minutes and one minute for immunization. During a revisit, history taking was assessed to take 5 minutes, the time for examination (8 minutes), drug administration (3 minutes), immunization (1 minute) and counselling (15 minutes) are the same as for the first visit. In addition, 3 minutes for the documentation of findings and 1 minute for welcoming the client were estimated per visit.

The more detailed analysis of the counselling process, where the time for each issue of counselling was clocked with a stopwatch, suggests an average time of 21 minutes if each listed issues is discussed. Table 4 shows the time spent separately for each subject of counselling. Since not all subjects of counselling in the check-list are to be covered on during each visit (see table 1), the average time for health education and counselling for a first visit and a revisit is in line with the 15 minutes assessed by the simulations. The detailed analysis showed furthermore that 2:40 minutes are required for providing information on drugs, supporting the estimation of 3 minutes based on the simulations. Overall standard ANC consultations according to the new model require 46 minutes for a first visit client and 35 minutes for a revisit client. Table 5 summarizes and compares the results of the time studies.

**Table 4:** Simulation of client education and counselling along focused ANC guidelines.

**Issue of counselling**	**Time (in min)**
Process of pregnancy and its complications	02:10
diet and nutrition	02:20
rest and exercise in pregnancy	01:00
personal hygiene	01:40
danger signs in pregnancy	03:00
use of drugs in pregnancy	01:00
effects of STI/HIV/AIDS	00:30
Voluntary counselling and testing for HIV*	01:00
Care of breasts and breast feeding	00:20
symptoms/signs of labour	01:10
plans of delivery	01:20
plans for postpartum care	00:30
family planning	03:20
harmful habits	00:20
schedule of return visit	00:20
time for any other questions	01:00

**TOTAL**	**21:00**

* Here, the client was only informed about the option of voluntary counselling and testing and was encouraged to accept the offer. No actual counselling and testing was undertaken

**Table 5:** Comparison of current performance and anticipated standard of focused ANC model

**Activity**	**Current practise**	**Focused ANC**	**Current practise**	**Focused ANC**
	first visit		revisit	
Registration	02:10	05:00	01:30	00:00
History taking	04:20	10:00	01:20	05:00
Examination	03:40	08:00	03:00	08:00
Drug administration	01:00	03:00	01:40	03:00
Immunization	01:40	01:00	01:00	01:00
Health education and counselling	01:30	15:00	00:00	15:00
*Total time direct activities*	*12:20*	*42:00*	*06:30*	*32:00*
Welcoming the client	01:00	01:00	01:00	01:00
Documentation of findings	02:00	03:00	01:30	03:00

**Total contact time**	15:20	46:00	09:00	36:00

## Discussion

The main findings of the study are: (1) Time requirements per consultation for the new model are substantially higher than current practise (2) The average time a health worker currently spends on an ANC first visit client is about 15 minutes, for a revisit client 9 minutes. (3) The major gap between current practise and the new model lies in health education and counselling.

Our finding of the 46 minutes time period for a first consultation of focused ANC corresponds well with the 40 minutes approximation made for the new WHO ANC model [5]. However, the estimate for the revisit differs: according to the WHO manual, a revisit should take 20 minutes, while this study suggests the need for 36 minutes. As there is no breakdown in the WHO manual for the time estimated for the different steps in the service provision, it is difficult to assess what components are responsible for the difference.

The impact, the required changes and the needed resources for the implementation of the new ANC model have been identified in the WHO randomised controlled trial. The trial was conducted in settings where 'resources available were sufficient for the implementation of adequate, basic, routine, western type antenatal care' [13]. The analysis compared the old ANC performance with the new model and it was concluded that the new model with reduced visits is as good as the old one, and even saves time and cost [5,13,14].

It is unlikely that such statements are also applicable for less developed countries like Tanzania, where current performance of ANC probably did not meet the definition of 'adequate, basic, routine western type ANC' and where – despite the official policy in the old model of recommended monthly visits, – the median number of ANC visits was already low (3,9 visits) [9]. Thus, the reduction of visits in ANC guidelines will not produce substantial savings in Tanzania. The implementation of the national adaptations of the new WHO ANC model can be expected to raise the standard of currently provided care: the identification of those in need of additional care will most probably increase. Focus on health education and counselling and the development of an individualized birth plan has the potential to increase the number of births attended by health professionals and antenatal referrals, recognized as one of the key factors to reduce maternal mortality [2].

Nevertheless, this study suggests that more than twice as much time as currently spent on ANC service provision is needed for implementing a good practise of the new model. It is thus questionable, if the new Focused ANC can be implemented without an increase of resources and in particular human resources.

The need for additional resources might become even more evident when ANC is used as an entry point for additional preventive interventions [2,4]. The new Focused ANC model in Tanzania already gives consideration to programmes such as nutrition and malaria, STIs and HIV/AIDS. However, if voluntary counselling and testing for HIV is to become part of ANC, additional 20 minutes per client will be required, as shown in a recent study at the Muhimbili University Hospital in Daressalaam, Tanzania [15]. The difference between current performance and Focused ANC including HIV voluntary testing and counselling would increase to 45 minutes. As literature confirms [16,17], the workload differs between dispensaries and between working days. Therefore, no general call for additional human resources for the implementation of the Focused ANC model seems adequate. While some dispensaries will be able to implement the new model with only some organisational improvements and adjustments, other settings will need additional staff. The challenge for health care managers will be to identify those in need of more resources.

We are aware that the current practise does not fully comply with the guidelines of the previous model. However, we want to highlight the change in resource requirements when changing from current practise to a meaningful implementation of the new model.

Health education and counselling has been identified as the major gap between the current performance and the proper performance of focused ANC. Information and communication are essential elements of health care provision. Reviews of women's experiences of maternity care highlight their importance [18]. Women participating in the new WHO model expressed their satisfaction with the nurses taking enough 'time to make them fully understand the message' [19,13]. The emphasis on ensuring that 'all women understand *why *they need to have a skilled attendant for their delivery, and *how *they are able to access obstetric care for emergencies' [3] is one of the most promising but at the same time most challenging components of the new ANC model.

Several issues need to be taken into account when interpreting our data on health education and counselling. Firstly, the general daily health talks were not included in our time analysis. Secondly, information given to the client during examination was not classified as counselling. Reassuring information on the current health status of the women and her pregnancy is valuable and important, but it is different from individual counselling, which refers to the specific conditions and preferences of the women and should lead to an individual birth plan, including contingencies for unforeseen events. The quality of counselling appears to be low. While the danger and prevention of anaemia and malaria as well as issues of hygiene and breastfeeding were the key topic of the information given, danger signs, the individual birth plan and birth preparedness were hardly mentioned. According to 1999 data, 6 in 10 women attending ANC in Tanzania had not received any information regarding pregnancy complications [8].

These findings highlight that so far no culture of counselling, in particular of individual counselling, has been developed. Bearing in mind that counselling, especially the development of an individual birth plan, and birth preparedness, is one of the major components of the new Focused ANC model, these findings suggest that much attention needs to be given to train on the concept of counselling, its importance and its requirements. This will require a learning process in providers and clients alike, as most women observed were rather passive and reluctant to ask questions. A close partnership between service provider and client is essential to take advantage of ANC as an entry point for behavioural change and awareness raising on danger signs.

Fifteen minutes of counselling for each visit is quite a long time, and the results of the simulation might suggest reconsidering the list of counselling items. While some health education issues such as diet and nutrition, exercise and rest during pregnancy, personal hygiene etc. can probably be addressed in group counselling, as practised in many African countries [20], the value of individual counselling in particular with regard to the development of an individualized birth plan and emergency preparedness needs to be highlighted.

## Conclusion

While the introduction of Focused ANC in Tanzania appears to have the potential to significantly improve the health of pregnant women and to increase the number of births with skilled attendants, such a change neither comes easily nor quickly. The programme is not just a small change in what was previously practised over many years, not just a reduction in the number of recommended visits, a focus on interventions proven to be effective and an appeal to abolish those practices that have been shown to be ineffective or harmful. In particular, with the focus on education and counselling as well as on individual counselling, the programme calls for a fundamental change in the attitudes, the skills and qualities required from ANC personnel.

Training that goes beyond the assimilation of knowledge of the pure facts of the content of each visit, but a training that fosters a deep understanding of the concept of focused ANC and thus tapping the roots of motivation, is essential to change old practices and to implement the new model.

Focused ANC is a great step forward. However, it will not come for free. This study suggests that the new model does not generally save cost and time on the provider side. Investment in additional human resources is necessary in some local settings to ensure its proper implementation. Given the expected benefits for maternal and perinatal health, it will be a very worthwhile investment.

## Competing interests

The author(s) declare that they have no competing interests.

## Authors' contributions

CvB, SF and AJ designed the study. AM and RM assisted in conducting the study in Tanzania. CvB analysed the data and drafted the manuscript. SF and AJ contributed to the interpretation of the data and helped in writing the paper. All authors read and approved the final manuscript.

## Pre-publication history

The pre-publication history for this paper can be accessed here:


